# Informatics Inference of Exercise-Induced Modulation of Brain Pathways Based on Cerebrospinal Fluid Micro-RNAs in Myalgic Encephalomyelitis/Chronic Fatigue Syndrome

**DOI:** 10.1089/nsm.2019.0009

**Published:** 2020-11-18

**Authors:** Vaishnavi Narayan, Narayan Shivapurkar, James N. Baraniuk

**Affiliations:** Division of Rheumatology, Immunology and Allergy, Department of Medicine, Georgetown University, Washington, District of Columbia, USA.

**Keywords:** micro-RNA (miRNA), cerebrospinal fluid, myalgic encephalomyelitis/chronic fatigue syndrome (ME/CFS), informatics, pathway analysis

## Abstract

**Introduction:** The post-exertional malaise of myalgic encephalomyelitis/chronic fatigue syndrome (ME/CFS) was modeled by comparing micro-RNA (miRNA) in cerebrospinal fluid from subjects who had no exercise versus submaximal exercise.

**Materials and Methods:** Differentially expressed miRNAs were examined by informatics methods to predict potential targets and regulatory pathways affected by exercise.

**Results:** miR-608, miR-328, miR-200a-5p, miR-93-3p, and miR-92a-3p had higher levels in subjects who rested overnight (nonexercise *n*=45) compared to subjects who had exercised before their lumbar punctures (*n*=15). The combination was examined in DIANA MiRpath v3.0, TarBase, Cytoscape, and Ingenuity software^®^ to select the intersection of target mRNAs. DIANA found 33 targets that may be elevated after exercise, including *TGFBR1*, *IGFR1*, and *CDC42*. Adhesion and adherens junctions were the most frequent pathways. Ingenuity selected seven targets that had complementary mechanistic pathways involving GNAQ, ADCY3, RAP1B, and PIK3R3. Potential target cells expressing high levels of these genes included choroid plexus, neurons, and microglia.

**Conclusion:** The reduction of this combination of miRNAs in cerebrospinal fluid after exercise suggested upregulation of phosphoinositol signaling pathways and altered adhesion during the post-exertional malaise of ME/CFS.

Clinical Trial Registration Nos.: NCT01291758 and NCT00810225.

## Introduction

Myalgic encephalomyelitis/chronic fatigue syndrome (ME/CFS) is a nociceptive, interoceptive fatiguing illness that is currently defined by symptoms and exclusion of other conditions in the extensive differential diagnoses.^1^ The etiology is unknown. Nociceptive refers to chronic pain and tenderness (systemic hyperalgesia and allodynia) that are controlled and regulated by spinal cord and brain processes.^2^ Interoception refers to the bodily sensations from internal organs and mucosal surfaces such as throat, bronchi (e.g., dyspnea^3^), gut, and lymph nodes.^4,5^ Fatigue refers to the cognitive and negative emotional strain involved in simple problem solving, loss of cognitive reserves related to insomnia and unrefreshing sleep, and influence of mitochondrial dysfunction and other mechanisms on muscular strength and stamina that combine to create a state of mental exhaustion and bodily heaviness.^6^ These principles are engrained in the 1994 Center for Disease Control criteria: moderate or severe, persistent, and sustained fatigue lasting more than 6 months and causing impairment of daily activities, plus moderate or severe complaints of at least four of eight ancillary criteria: short-term memory or problems with concentration, sore throat, sore lymph nodes, myalgia, arthralgia, sleep disturbances, new-onset headaches that include migraine, and post-exertional malaise. Post-exertional malaise, also referred to as exertional exhaustion, is a unique characteristic of ME/CFS.^7^

We modeled post-exertional malaise by having subjects perform two submaximal exercise stress tests on consecutive days.^8^ Functional magnetic resonance imaging during a working memory task was compared between pre-exercise and post-exercise time points and demonstrated exercise-induced cognitive dysfunction. Because the neurological symptoms suggested brain pathologies, lumbar punctures were performed after exercise to sample the cerebrospinal fluid (post-exercise ***CFS*** group). Lumbar punctures were also performed in a separate group who had rested overnight and did not have exercise (nonexercise ***cfs0*** group). Cerebrospinal fluid specimens were assayed for micro-RNAs (miRNA),^9^ proteomics,^10^ metabolomics, and other analytes to interrogate the central neurotoxic pathologies proposed in ME/CFS.^11–13^ ***CFS*** and ***cfs0*** were compared in a cross-sectional manner for these pioneer studies to assess exercise-induced differences in miRNA expression.

miRNAs are ∼22 nucleotide-long, single-stranded RNAs transcribed from genomic DNA.^14^ The primary miRNA is transcribed from intergenic DNA or introns of mRNAs. Pre-miRNAs are processed by the RNase protein DROSHA in the nucleus before export into the cytoplasm for processing by DICER protein.^15^ The miRNA is loaded onto the Argonaute protein(s) to form the RNA-induced silencing complex that can then bind to complementary sequences in the 3′ untranslated region of target mRNAs to repress translation or promote mRNA degradation. miRNAs dynamically fine-tune the expression of mRNAs, their translated proteins, and hence signaling and other pathways.^16^ Relatively elevated miRNA levels have greater binding to target mRNAs leading to destruction of the miRNA-mRNA duplex and loss of protein and pathway activities. Conversely, relatively diminished miRNA levels allow unobstructed mRNA translation and elevation of the protein and pathway functions. A single miRNA may target anywhere from zero to over a thousand mRNAs. Most studies investigate the effects of single miRNAs, but not the synergistic or antagonistic effects that may occur when a combination of miRNAs are modulated simultaneously.

We hypothesized that exercise would significantly alter the levels of miRNAs in ME/CFS subjects, and that informatics analysis of the downstream targets would allow inferences about the mRNAs, proteins, and pathways that were modulated. The functional effects may allow inferences about potential mechanisms of post-exertional malaise. Quantitative polymerase chain reaction (qPCR) was used to measure cerebrospinal fluid miRNAs in specimens from nonexercise (***cfs0***) and post-exercise ***CFS*** subjects.^9^ Five miRNAs were found to have significantly higher levels in ***cfs0*** than ***CFS*** subjects (***cfs0***>***CFS*** condition). Because each of the miRNAs may target hundreds of miRNAs, we developed an informatics strategy to search for targets based on combinations of miRNAs and the intersection of genes and their pathways in the DIANA search engine.^17^ This method was an alternative to searching for individual lists and then creating the union of all potential targets in the literature and databases derived from text mining. The list of miRNAs was assessed in several online databases to determine target pathways, downstream protein interactions, and potential target cells and brain regions that may be relevant to pathologies of ME/CFS and post-exertional malaise.

## Materials and Methods

### Clinical information

All subjects gave written informed consent to protocols approved by the Georgetown University Institutional Review Board (IRB 2009-229, 2013-0943, and 2015-0579) and the Human Research Protection Office of the Department of Defense Congressionally Directed Medical Research Program (HRPO A-15547 and A-18479), and listed in clinicaltrials.gov. The studies followed World Medical Association Declaration of Helsinki—Ethical Principles for Medical Research Involving Human Subjects.^18^ The investigations were not considered clinical trials using the World Health Organization (WHO) definition.^19^

CFS was diagnosed during history and physical examinations with inclusion according to the 1994 Center for Disease Control criteria^7^ and exclusion for chronic medical and psychiatric diseases.^20,21^ Subjects completed the CFS Symptom Severity Questionnaire and reported the severity of their fatigue and the eight ancillary criteria of poor memory or concentration, sore throat, lymph nodes, muscle pain, joint pain, headaches, sleep, and exertional exhaustion^7^ using an ordinal system with 0 for no symptom, 1 for trivial severity, 2 for mild, 3 for moderate, and 4 for severe complaints.^22^

The nonexercise group rested overnight before lumbar puncture (nonexercise, ***cfs0***, *n*=45).

The second group had submaximal bicycle exercise stress tests on 2 consecutive days before lumbar puncture (***CFS***, *n*=15).^9^

### qPCR assay

RNA was extracted from 0.5 mL of cerebrospinal fluid, and miRNAs profiled by qPCR with a 380-miRNA panel as described previously.^9^ miRNA levels were quantified from cycle thresholds (Ct) using the ΔΔCt method.^23,24^ Data were refined for this analysis by using a normalizer that was the average Ct from 11 miRNAs that were detected in all specimens and were not significantly different between groups. The averaged normalizer Ct value was subtracted from the Ct of each miRNA to calculate ΔCt for each individual. ΔCts were averaged for each group. ΔΔCt for each miRNA was computed as the difference between non-exercise (***cfs0***) and post-exercise (***CFS***) group ΔCt values with positive values indicating high miRNA levels in the ***cfs0***>***CFS*** condition. Statistical significance was found using analysis of variance (ANOVA) to assess ***cfs0***, ***CFS***, and control subject nonexercise and post-exercise ΔΔCt samples, followed by Tukey Honest Significant Difference (HSD, *p*<0.05) and false discovery rate (FDR, *p*<0.05) to correct for multiple comparisons. Five miRNAs were significantly higher in the nonexercise than post-exercise group (miRNA higher in ***cfs0***>***CFS*** condition). Receiver operating characteristics (ROC) to distinguish ***cfs0*** from ***CFS*** were determined for each miRNA.

### Target analysis

**FIG. 1.**
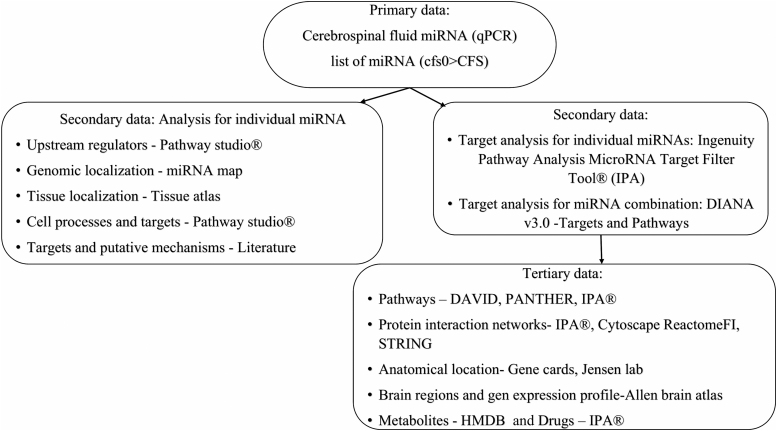
Informatics workflow explaining the flow of miRNA data from qPCR to targets and pathways. miRNA, micro-RNA; qPCR, quantitative polymerase chain reaction.

Lists of target genes were defined using combinations of miRNAs in DIANA mirPathv3.0 (http://snf-515788.vm.okeanos.grnet.gr/)^17^ and single miRNAs in Pathway Studio^®^ (https://mammalcedfx.pathwaystudio.com/login/form),^25^ Ingenuity Pathway Analysis (IPA) MicroRNA Target Filter Tool^®^ (https://www.qiagenbioinformatics.com/products/ingenuity-pathway-analysis/),^26^ and by literature searches (Fig. 1). The DIANA search process with genes intersection was preferred because it searched for genes that interacted with combinations of miRNAs. Single miRNAs interact with zero to thousands of mRNAs from multiple pathways. The gene union and pathway union and intersection searches from separate miRNA searches led to long lists of gene interactions, but did not provide information about potential additive or synergistic effects resulting from the combinations of miRNA changes.

The combination of miRNAs was entered into DIANA miRpath v3.0 software (http://diana.imis.athena-innovation.gr/DianaTools/index.php?r=microT_CDS/index)^17^ and DIANA-TarBase v7.0 (http://carolina.imis.athena-innovation.gr/diana_tools/web/index.php?r=tarbasev8/index)^17^ database to identify the gene intersection (*p*<0.001) from KEGG (Kyoto Encyclopedia of Genes and Genomes; https://www.genome.jp/kegg/)^27^ and GO (Gene Ontology; http://geneontology.org/)^28^ databases using the highest number of miRNAs for each search.^17^ Gene intersection selected genes that had interactions with multiple miRNAs. This process restricted the total number of genes selected because genes with multiple miRNA interactions were favored. By comparison, gene union provided the combination of all genes from all five miRNAs and did not preferentially select genes influenced by more than one of the five miRNAs. Pathway union and pathway intersection were not used because they also selected large numbers of genes without direct information about miRNA-mRNA interactions.

The DIANA outcomes were lists of pathways and candidate mRNAs that were targeted by combinations of miRNAs in KEGG and GO. A threshold of *p*<0.001 was applied to restrict our gene—miRNA interactions and pathways to those that were most likely to operate *in vivo*. To select candidate mRNAs that were most likely to be modulated by the combination of miRNAs, all candidates were weighted by the predicted number of miRNAs they interacted with (#miRNA) and probability (*p*) of the regulatory pathway provided by DIANA:

$$Weight=\left(\#miRNA\right)\times \left(-1\right)\left(log_{10}p\right).$$

A novel MATLAB script was used to rank candidate mRNAs according to the sum of weight for each gene (Σweight) and the number of times a gene (Σhits) appeared in the different pathways (Supplementary Tables S2–S5). The weighting process selected 19 genes from KEGG and 16 from GO categories. Results were combined and reconciled to identify the final target gene list of 33 mRNAs.

The IPA MicroRNA Target Filter Tool (https://www.qiagenbioinformatics.com/products/ingenuity-pathway-analysis/)^26^ analyzed the combination of five miRNAs simultaneously and found seven additional experimentally validated targets.

Individual miRNAs were searched in tarbase v8.0 and microTCDS from DIANA, and in Pathway Studio (https://mammalcedfx.pathwaystudio.com/login/form)^25^ using its proprietary database and algorithm to identify a separate list of target genes (Supplementary Tables S11 and S12).

Literature searches for each individual miRNA found an additional 18 experimentally validated targets.

### Pathway analysis

In some cases, the DIANA outcomes selected general terms like “organelle” (GO:0043226, 16 genes), “nucleoplasm” (GO:0005654, 8 genes), “Transcriptional misregulation in cancer (hsa05202),” “Adherens junction,” and “Chronic myeloid leukemia (hsa05220)” that provided few mechanistic insights. Therefore, we used an iterative refinement strategy to search for more specific mechanisms and pathways using other databases. DAVID Bioinformatics Resources 6.8 (https://david.ncifcrf.gov/summary.jsp)^29^ (Enrichment Score >2, Benjamini Hochberg *p*<0.05) and Reactome (https://reactome.org/)^30^ generated lists of pathways that were significantly enriched for the target genes (*p*<0.001). Pathway Studio^25^ provided upstream regulators and the top 5 biological processes. Panther (www.pantherdb.org/)^31^ gave the top enriched GO terms for the target genes (*p*<0.05). IPA software^®26^ generated a protein interaction network map with linkers, and a list of enriched pathways. ReactomeFI app (https://reactome.org/tools/reactome-fiviz)^32^ was downloaded into Cytoscape (https://cytoscape.org/)^33^ and used to cluster the targets into modules according to pathway enrichment. Modules were ranked by the largest number of clustered gene targets. The top pathway was identified for each module. The protein–protein association network was drawn by STRING v:11.0. (https://string-db.org/)^34^The iterative process honed our outcomes from general terms into smaller segments of large pathways and subsets of general GO terms.

### Cellular and tissue localization

Genomic localization and parent genes for miRNAs were obtained using miRNA Map (http://mirnamap.mbc.nctu.edu.tw/),^35^ UCSC genome browser (https://genome.ucsc.edu/cgi-bin/hgGateway),^36^ and Entrez Gene (https://www.ncbi.nlm.nih.gov/gene)^37^ databases.

Tissue Atlas for miRNA (https://ccb-web.cs.uni-saarland.de/tissueatlas/)^38^ found tissues that were enriched for each individual miRNA.

The Allen Institute Brain Atlas (http://human.brain-map.org/) mapped the anatomical distribution of the brain transcriptome mRNAs detected by *in situ* hybridization to six normal *ex vivo* post-mortem brains.^39^ mRNA levels were compared to the overall brain average to define brain regions where each mRNA was significantly upregulated or downregulated, or equivalent to the rest of the brain.^40,41^

We previously found the cerebrospinal fluid miRNA levels were equivalent for nonexercise control and ***cfs0*** groups.^9^ Therefore, we assumed that the expression in the nonexercise ***cfs0*** group of volunteers was similar to the *ex vivo* post-mortem brains in the Allen atlas. Because cerebrospinal fluid is generated in the choroid plexus that is located in the lateral ventricles, we extracted transcriptomes of these structures from the Allen atlas and Jensen (https://tissues.jensenlab.org/Search)^42^ databases and relevant publications^43^ to create a choroid plexus-enriched transcriptome. Gene cards (https://www.genecards.org/)^44^ were used to find the cellular localization for the target proteins in neurons, microglia, choroid plexus, and other brain cells (Fig. 1).

## Results

### Demographics

At baseline, the nonexercise (***cfs0***) and post-exercise (***CFS***) groups were equivalent for all demographic measures, severity of CFS complaints^7,22^ and frequency of fibromyalgia by 1990 criteria^45^ (Table 1).

**Table 1.** Demographics

Groups	cfs0	CFS
Exercise	No exercise	Submaximal test
*N*	45	15
Age	45.7±11.0	45.0±10.16
Female	80%	60%
Body mass index	28.7±7.18	27.3±5.79
Fibromyalgia 1990	*N*=18/45 (40%)	*N*=4/15 (27%)
Dolorimetry (kg)	2.81±1.48	4.04±2.14
CFS symptom severity scores		
Fatigue	3.69±0.52	3.71±0.61
Memory	3.10±0.79	2.79±0.70
sore_throat	1.43±1.23	1.64±1.01
lymph nodes	1.33±1.24	1.07±1.14
muscle_pain	2.98±1.18	2.50±1.29
joint_pain	2.50±1.23	2.21±1.37
Headaches	2.50±1.29	1.64±1.28
Sleep	3.50±0.80	3.43±0.65
Exertion	3.38±0.91	3.50±0.52

There were no significant differences in demographics, fibromyalgia, or severity of CFS complaints (average±SD) between the non-exercise (***cfs0***) and post-exercise (***CFS***) groups.

CFS, chronic fatigue syndrome.

### Cerebrospinal fluid miRNAs and target genes

miR-608, miR-328, miR-200a-5p, miR-93-3p, and miR-92a-3p had higher levels in cerebrospinal fluid in the nonexercise ***cfs0*** than post-exercise ***CFS*** group (Table 2). Therefore, ***CFS*** had diminished miRNA levels that presumably promoted the elevation of mRNA translation, protein synthesis, and cellular function after exercise. These 5 miRNAs met our criteria for ΔΔCt ≥2-fold change, plus significant ANOVA, HSD, and FDR (*p*<0.05) (Supplementary Table S1) using the 11 miRNA normalizer (Supplementary Table S1 and Supplementary Fig. S1). miR-let-7i-5p was excluded because it did not meet these criteria (ΔΔCt=1.91±2.25, mean±SD, HSD=0.034, and FDR=0.080).

**Table 2.** Relative miRNA Levels in *cfs0>CFS* After Normalization of Quantitative Polymerase Chain Reaction Using 11 miRNAs (Mean±SD)

	ΔΔCt for ***cfs0***>***CFS*** (mean±SD)	Receiver operating characteristics			
		AUC	p	Sensitivity	Specificity
miR-328	4.61±3.67 HSD=0.000 FDR=0.0013	0.877	0	0.80	0.80
miR-608	2.85±2.43 HSD=0.001 FDR=0.0037	0.819	0	0.80	0.778
miR-200a-5p	2.53±2.16 HSD=0.001 FDR=0.0036	0.837	0	0.80	0.80
miR-92a-3p	2.29±2.06 HSD=0.008 FDR=0.0070	0.804	0	0.80	0.778
miR-93-3p	2.10±2.03 HSD=0.001 FDR=0.015	0.818	0	0.733	0.733

Differences were significant if ANOVA *p*<0.05, Tukey HSD <0.05, FDR <0.05, and ROC asymptotic significance <0.05 (Supplementary Fig. S1).

ANOVA, analysis of variance; AUC, area under the curve; FDR, false discovery rate; HSD, honest significant difference; miR, micro-RNA; ROC, receiver operating characteristics.

### Genomic localization of miRNA

Genomic localization of the five miRNAs and their parent genes was relevant for understanding miRNA expression and regulation. miR-328 and miR-608 are located in introns of ELMO3 (engulfment and cell motility protein 3, chromosome 16q22.2) and SEMA4G (semaphorin-4G gene, 10q24.31), respectively. miR-93-3p was unusual because it was clustered with miR-106 and miR-25 in intron 13 of the *MCM7* (minichromosome maintenance complex component 7, 7q22.1) gene indicating tight regulation between miR-93-3p and *MCM7*.^46^ miR-92a-3p (13q31.3) is hosted by the MIR-17-92a-1 Cluster Host Gene. miR-200a-5p (1p36.33) is intergenic.

### Upstream regulators for the miRNAs

Pathway Studio^25^ found complex regulation of miRNA expression. miR-93 was elevated by HDAC and MYC, diminished by LEP and AKT1, and modulated in unknown manner by TGFB1 and DICER. miR-200a was diminished by TGFB1, elevated by DICER, and also influenced by LEP and HDACs. miR-92a was modulated by TGFB1, AKT1, MAPK, and MYC. Additional interactions have been proposed, but effects were not documented (Supplementary Table S9).

### Target genes

#### DIANA miRpath

The combination of five miRNAs was used to search for gene targets using TarBase v7.0 and DIANA v3.0 and gene intersection. The intersection of the five miRNAs was used because individual miRNAs identified many target genes ranging from 1970 (miR-92a-3p), 883 (miR-93-3p), 175 (miR-200a-5p), and 49 (miR-608) to 0 (miR-328). The union of these genes was not manageable for defining the effects of the co-expressed miRNAs and may have identified targets and pathways that occurred only once out of the hundreds of targets found in the searches. KEGG and GO databases were searched using the highest number of miRNAs possible to select pathways with *p*<0.001. The significant KEGG pathways found using four miRNAs were *Adherens junction (hsa04520)* and *Transcriptional Misregulation in Cancer (hsa05202)*. Each pathway contained seven target genes. Search of the GO category database with four miRNAs identified *organelle* (*GO:0043226*, 16 genes) and *nucleoplasm* (*GO:0005654*, 8 genes) (Supplementary Tables S6 and S7). Potential target genes were weighted (Supplementary Methods) and the KEGG and GO lists reconciled to identify 33 targets. Initial inspection was noteworthy for the receptors *TGFBR1*, *IGF1R*, and *SCARB2*, cell cycle proteins *CCND2*, *CCNE2*, and *CDC42*, and transcription factors *ELK4*, *TCF3*, and *ZNF703* (Table 3). For clarity, the DIANA gene targets are shown in italics.

**Table 3.** Target Gene List (*n*=33) for the Intersection of Five miRNAs Using DIANA mirPath v3.0 for the ***cfs0***>***CFS* Condition (“The DIANA list”) Organized by Function**

Gene symbol	Protein name	Chromosome location
*Adherens junction hsa04520*		
*ACTB*	actin beta	7p22.1
*CDC42*	cell division cycle 42	1p36.12
*DYNC1H1*	dynein cytoplasmic 1 heavy chain 1	14q32.31
*FYN*	FYN proto-oncogene, Src family tyrosine kinase	6q21
*IGF1R*	insulin like growth factor 1 receptor	15q26.3
*IQGAP1*	IQ motif containing GTPase activating protein 1	15q26.1
*PTPRJ*	protein tyrosine phosphatase, receptor type J	11p11.2
*TGFBR1*	transforming growth factor beta receptor 1	9q22.33
*RNA binding GO:0003723*		
*DCP2*	decapping mRNA 2	5q22.2
*EWSR1*	EWS RNA binding protein 1	22q12.2
*HNRNPC*	heterogeneous nuclear ribonucleoprotein C (C1/C2)	14q11.2
*NCL*	nucleolin	2q37.1
*NUFIP2*	nuclear FMR1 interacting protein 2	17q11.2
*PCBP2*	poly(rC) binding protein 2	12q13.13
*PRPF8*	pre-mRNA processing factor 8	17p13.3
*Transcription, DNA templated GO:0006351*		
*ELK4*	ETS transcription factor	1q32.1
*KMT2D*	lysine methyl transferase 2D	12q13.12
*SETD7*	SET domain containing lysine methyl transferase 7	4q31.1
*SIN3A*	SIN3 transcription regulator family member A	15q24.2
*TCF3*	transcription factor 3	19p13.3
*ZNF703*	zinc finger protein 703	8p11.23
*Nucleoplasm GO:0005654*		
*ASH1L*	ASH1 like histone lysine methyltransferase	1q22
*BAG6*	BCL2 associated athanogene 6	6p21.33
*CCND2*	cyclin D2	12p13.32
*CCNE2*	cyclin E2	8q22.1
*DYRK1A*	dual specificity tyrosine phosphorylation regulated kinase 1A	21q22.13
*H3F3B*	H3 histone family member 3B	17q25.1
*MCM7*	minichromosome maintenance complex component 7	7q22.1
*SUV420H1*	lysine methyl transferase 5B	11q13.2
Other		
*FASN*	fatty acid synthase	17q25.3
*SCARB2*	scavenger receptor class B member 2	4q21.1
*SCD*	stearoyl-CoA desaturase	10q24.31
*SPOPL*	speckle type BTB/POZ protein like	2q22.1

The tarbase v8.0 tool^47^ was used for separate searches with each of the five miRNAs to find the miRNA-gene interactions. Using prediction scores=1, 18 gene interactions were found for the five miRNAs (Supplementary Table S11). These genes were searched in DAVID and identified *positive regulation of ubiquitin protein transferase activity* (*p*=0.019), *protein binding* (*p*=0.026), *DNA damage* (*p*=0.033), and *phosphoprotein* (*p*=0.035). *ELK4*, *HNRNPC*, *IGF1R*, and *SETD7* matched between the tarbase v8.0 and DIANA miRpath targets. *DKK3*, *ITGA5*, and *MAP2K4* matched between the tarbase and IPA lists. None matched the literature search list.

The microTCDS tool^48^ in DIANA tools examined individual miRNA interactions. Using a miTG score >0.95 revealed 153 gene targets, 17 targets for miR-328, 48 targets for miR-608, 27 targets for miR-200a-5p, 30 targets for miR-92a-3p, and 49 targets for miR-93-3p (Supplementary Table S12). However, none of these targets matched with the DIANA miRpath list. *ADRB1*, *ITGA5*, and *MAP2K4* matched the IPA list. None matched the literature search list. Pathways found with DAVID were *transcription factor activity* (*p*=0.022), *DNA binding* (*p*=0.037), *zinc finger* (*p*=0.018), and *nucleus* (*p*=0.038).

#### Pathway Studio

The five miRNAs were searched in individual manner using Pathway Studio and its proprietary text mining and database methods. The union of the five searches identified 42 target genes that did not overlap with the DIANA list (Table 4).

**Table 4.** MicroRNA Targets from Pathway Studio

miRNA	Positive (target elevated)	Negative (target inhibited)	Unknown effect
miR-200a	PI3K, CD36, IL6, TNF, IL1A, PAX6, CASP3, HMOX1, CDKN1A, glutathione transferase(s)	CDH2, WNT, SP7, VIM, ACTA2, PKD1, collagen(s)	NOTCH1, VEGFA, BCL2L11
miR-328	Collagen(s)	MYC, CD36, CASP3	VIM, PTEN, CCND1
miR-608		IL6, MAPK1, AKT1, ERBB2	PI3K, CD44, MAPK8,
miR-92a	PI3K, ACTA2	IL6, TNF, COL1A1, JUN	TMP3, COL2A1, MMP9, AKT1
miR-93	ABCG2, MYC	VIM, SMAD2, MMP9, CDH2, ITG, TP53, BCL2L1	PI3K, FN1, CD274, CDH1, inflammatory cytokine(s)

Positive regulation indicated the target genes were elevated by the miRNA. Negative regulation suggested direct inhibition of the mRNA to diminish the target protein expression. Interactions with unknown outcomes may increase or decrease target mRNA levels.

PI3K was predicted to be elevated by miR-200a and miR-92a (positive, indirect effect), with unknown effects for miR-608 and miR-93. IL6 was reported to be elevated by miR-200a, but inhibited by miR-608 and miR-92a (negative, probably direct inhibition of IL6 target mRNA). *VIM* was inhibited by miR-200a and miR-93, with an unknown effect by miR-328. CD36 was elevated by miR-200a, but diminished by miR-328. Collagens were affected in opposite directions. TNF was elevated by miR-200a and diminished by miR-92a. Additional targets were selected by individual miRNAs. However, it is not always apparent if the miRNAs bound directly to the 3′ UTR of these genes. The Pathway Studio union list did not overlap the list selected using intersection of pathways and miRNAs in DIANA. Therefore, the target list from DIANA was used for further analysis. Pathway Studio outcomes were considered to be parallel or complimentary findings.

Literature searches identified 22 potential targets with evidence of direct miRNA binding to 3′ UTRs. Only *CDC42* was shared with the DIANA list. General categories were adhesion and extracellular matrix (MMP16, CD44, CDC42, COL5A1, ZEB1, ZEB2, CTNNB, ADAMTS4, and ADAMTS5). miR-200a-5p modulated PTEN, which recycles inositol-trisphosphate for the *PIK3R3-AKT* pathway (Table 5).^49–65^

**Table 5.** Literature Search Results for Individual miRNAs and Downregulated Target Genes

miRNA	Genes	Name
miR-328	SFRP1 (49)	Secreted Frizzled-related protein 1
	ABCG2 (59)	ATP binding cassette subfamily G2
	MMP16 (57)	Matrix metalloproteinase 16
	PIM-1 (29)	Pim-1 proto-oncogene, serine/threonine kinase
	CD44 (30)	CD44 molecule Indian blood group
	SLC2A1/GLUT1 (31)	Solute carrier family 2 member 1/glucose transporter
miR-608	ACHE (24)	Acetylcholinesterase
	CDC42 (24)	Cell division control protein 42
	IL6 (24)	Interleukin 6
	RRM1 (37)	Ribonucleotide reductase catalytic subunit M1
	CDA (37)	Cytidine deaminase
	COL5A1 (36)	Collagen 5A1
miR-200a-5p	ZEB1 and ZEB2 (33)	Zinc finger E-box-binding homeobox 1 and 2
	CTNNB1 (32)	β-catenin
	PTEN (35)	Phosphatase and tensin homolog
miR-92a-3p	ADAMTS-4 and ADAMTS-5 (38)	ADAM metallopeptidase with thrombospondin type 1 motif and type 5 motif
	HDAC2 (37)	Histone deacetylase 2
	WNT5A (39)	Wingless type MMTV integration site family member 5
miR-93-3p	ULK1 (26)	Ubiquitin-linked kinase 1
	CAPN2 (28)	Calpain-2

#### IPA MicroRNA Target Filter

**FIG. 2.**
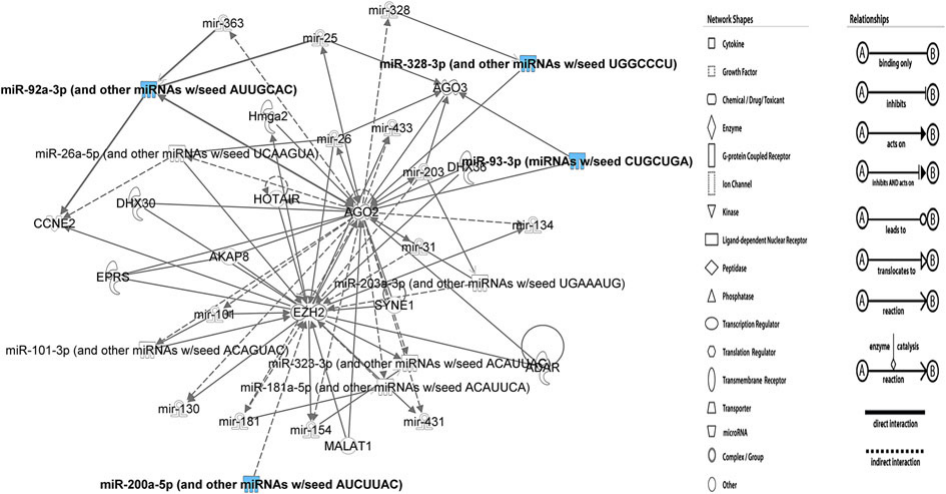
IPA MicroRNA Target Filter^®^ Network formed with three miRNAs miR-92a-3p, miR-328-3p, and miR-200a-5p.

The five miRNAs were searched as a group with IPA MicroRNA Target Filter tool. Experimentally validated and highly predicted targets were found for miR-92a-3p (*n*=191) and 200a-5p (*n*=40). The mRNA targets and predicted pathways were redundant with, for example, 13 genes shared between *cardiac hypertrophy signaling* and *molecular mechanisms of cancer* and 7 with *Wnt signaling*. Conversely, PIK3R3 was the most prevalent mRNA as it was matched with 175 pathways. Many of these pathways were named for upstream mediators and their receptors that used PIK3R3 for downstream intracellular signaling. The lists were consolidated to seven targets (Table 6) that were fed into DAVID for further iterative pathway analysis (Supplementary Table S8). The IPA Network showed that EZH2, a histone methyl transferase, and AGO2 had the most protein interactions (connections) (Fig. 2). *CCNE2* was shared with the DIANA list.

**Table 6.** IPA Top Ranked Targets

ADCY3	Adenylate cyclase type 3
CAMK2A	Calcium-/calmodulin-dependent protein kinase type II subunit alpha
GNAQ	Guanine nucleotide-binding protein G(q) subunit alpha
MAP2K4	Dual-specificity mitogen-activated protein kinase 4
PIK3R3	Phosphatidylinositol 3-kinase regulatory subunit gamma
RAP1B	Ras-related protein Rap-1b
RAP2A	Ras-related protein Rap-2a

### Pathway analysis

#### Pathways predicted for DIANA target genes

The 33 targets from DIANA (Table 3) were iteratively fed into other search engines to refine the selection of relevant pathways.

Pathway enrichment in DAVID identified *Adherens junction*, *RNA binding*, and *Nucleosome-RNA polymerase complex mechanisms*.^29^

In PANTHER,^31^ the target genes were enriched for GO terms related to *epidermal growth factor responses*, *histone methylation*, *dendritic spine organization*, and *viral RNA genome replication*.

IPA using the IPA Core analysis tool^®26^ selected *Molecular Mechanisms of Cancer* (*p*=0.0000016), *Epithelial Adherens Junction Signaling* (*p*=0.0000025), *Cyclins and Cell Cycle Regulation* (*p*=0.00024), and *TGF Signaling* (*p*=0.0081) as its top hits.

#### Protein interaction networks

**FIG. 3.**
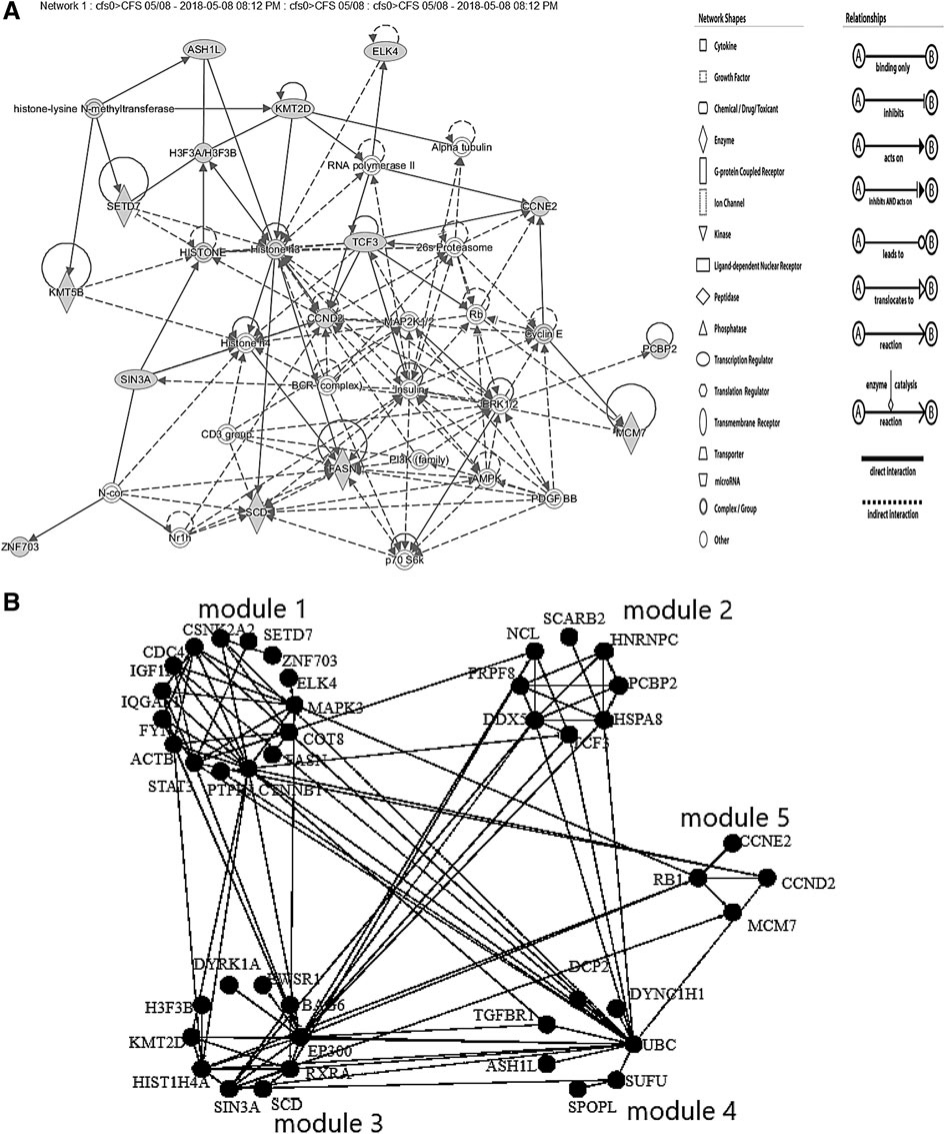

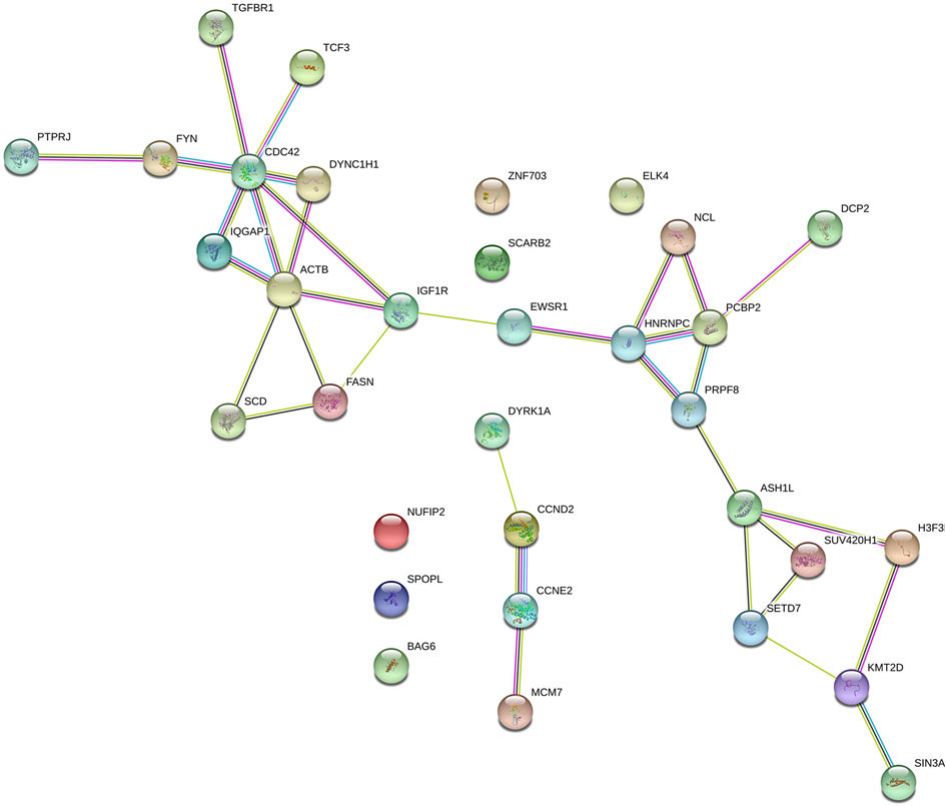
Target protein interaction networks. The top networks that included linker proteins were drawn with **(A)** IPA and **(B)** Cytoscape ReactomeFI. **(C)** STRING showed networks without linkers. IPA, Ingenuity Pathway Analysis^®^.

The gene targets from DIANA were organized into protein interaction networks using IPA, Cytoscape ReactomeFI, and STRING. The top IPA network contained 14 target genes plus linkers that matched the Reactome pathway *PKMTs methylate histone lysine (R-HSA-3214841)* (Fig. 3A). The second IPA network matched *Signaling by Receptor Tyrosine Kinases (R-HSA-9006934).*

The Cytoscape Pathway Enrichment Algorithm and ReactomeFI app organized the DIANA target genes into five modules by incorporating linker proteins: *Adherens junction (hsa04520)*, *Beta integrin cell surface interactions (NCI pathway interaction database)*, *Processing of capped intron-containing pre-mRNA (R-HSA-72203)*, and *Cell cycle (hsa04110)* (Fig. 3B and Table 7).

**Table 7.** Cytoscape Modules of Target Proteins with Linkers using ReactomeFI App

Rank	Modules of target proteins with linkers	Top pathway for each module
1	*ACTB*, *CDC42*, *ELK4*, *FASN*, *FYN*, *IGF1R*, *IQGAP1*, *PTPRJ*, *SETD7*, *ZNF703*	*Adherens junction (KEGG hsa 04520)*
	(Linkers: CCT8, CSNK2A2, CTNNB1, MAPK3, STAT3)	
2	*BAG6*, *DYRK1A*, *EWSR1*, *H3F3B*, *KMT2D*, *SCD*, *SIN3A*	*Transcriptional misregulation in cancer (KEGG hsa05202)*
	(Linkers: EP300, HIST1H4A, RXRA)	
3	*DDX5*, *HNRNPC*, *NCL*, *PCBP2*, *PRPF8*, *SCARB2*, *TCF3*	*Processing of capped intron-containing pre-mRNA (Reactome R-HSA-75067)*
	(Linker: HSPA8)	
4	*ASH1L*, *DCP2*, *DYNC1H1*, *SPOPL*, *TGFBR1*	*Beta5 beta6 beta7 and beta8 integrin cell surface interactions (NCI PID)*
	(Linkers: SUFU, UBC)	
5	*CCND2*, *CCNE2*, *MCM7*, *RB1*	*Cell cycle (KEGG hsa04110)*

STRING showed the interactions between the target proteins without linkers. The result was a long-extended network with several unconnected genes. STRING identified *Adherens junction (hsa04520)* with a subnetwork centered by *CDC42* and *ACTB* (*ACTB*, *CDC42*, *FYN*, *IGF1R*, *IQGAP1*, *PTPRJ*, and *TGFBR1*) (Fig. 3C upper left), *Lysine degradation* (*hsa00310*: *ASH1L*, *KMT2D*, *SETD7*, and *SUV420H1*) (Fig. 3C lower right), and *Transcriptional misregulation in cancer* (*hsa05202*: *CCND2*, *ELK4*, *EWSR1*, *IGF1R*, *SIN3A*, amd *TCF3*).

#### Pathway analysis from targets of IPA MicroRNA Target Filter

**FIG. 4.**
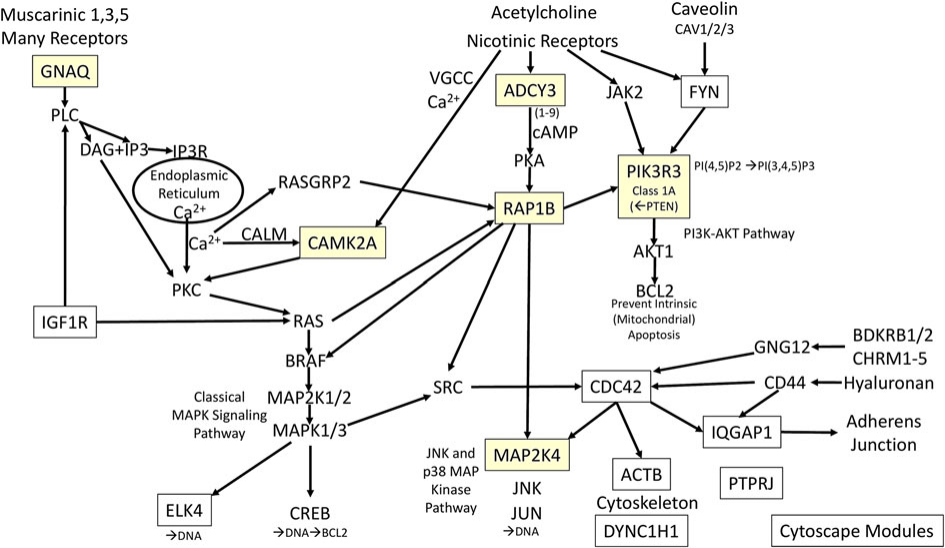
Compilation of KEGG pathways from DIANA and IPA^®^ target lists. Targets found by searching through DIANA were in white boxes, while IPA targets were highlighted in light yellow. Representative members of PLC, PKC, cAMP, PIK3R3–AKT, Adherens junction, and classical MAPK and MAPK–JNK were interconnected. Cytoscape classified 15 other DIANA targets to adhesion and integrin pathways (Table 6). KEGG, Kyoto Encyclopedia of Genes and Genomes.

The seven targets from IPA MicroRNA Target Filter were searched in DAVID and the top KEGG pathways were found. The KEGG pathway diagrams were inspected and cross-referenced for redundant proteins in signaling pathways. This process identified potential upstream mediators, seven transmembrane G protein-coupled receptors and their downstream signaling mechanisms. Acetylcholine was one prominent mediator because its muscarinic and nicotinic receptors acted through several signaling pathways (*hsa04725 Cholinergic synapse*) (Fig. 4).

GNAQ was one hub that linked muscarinic receptors M1, M3, and M5 (CHRM1/3/5) and many other mediators and receptors to activation of phospholipase C (PLC), diacylglycerol, and inositol trisphosphate production, endoplasmic reticulum calcium release, activation of calmodulin and CAMK2A, protein kinase C, and the classical MAPK pathway (*MAPK signaling pathway hsa04010)*. GNAQ was activated by other agonists, including angiotensin, cholecystokinin, gonadotrophin-releasing hormone, thrombin, trypsin, oxytocin, adenosine, lysophosphatidic acid, cysteinyl leukotrienes, adrenaline, and noradrenaline that acted by binding to their seven transmembrane receptors, including LPAR1 through 5, ADORA2B, ADORA2A, F2R, F2RL3, P2RY1, FPR1, PTGFR, OXTR, GRPR, GRM5, CYSLTR1, CYSLTR2, CCKAR, and CCKBR. The PLC pathway was also activated by *IGF1R*.

Acetylcholine also acted through nicotinic receptors to activate four other signaling mechanisms. Adenylyl cyclase 3 (ADCY3) generates cAMP that activates PKA and RAP1B (*RAP1 signaling pathway hsa04010)* followed by MAPK and JNK pathway (*JNK and p38 MAP kinase pathway hsa04010*). Calcium flux through the voltage-gated calcium channel pathways activates CAMK2A. Activation of *FYN* and JNK stimulates the PIK3R3 and AKT pathway (*PI3K and AKT pathway hsa04151*).

Cholinergic CHRM1/3/5 receptors also are linked to the G protein GNG12 that activates *CDC42* and provides signaling links to *IQGAP1* and *Adherens junction*, *actin and cytoskeleton*, *and MAPK and JNK pathways*. The target lists and pathways found using the DIANA search and IPA miRNA Target Filter were different, but complementary for identifying pathways modified by GNAQ, *IGF1R*–PLC–MAPK pathways, ADCY3–RAP1B–MAPK JNK pathways, *FYN*–PIK3R3–AKT, and *CDC42*–*IQGAP1*–Adherens junction interactions.

**FIG. 5.**
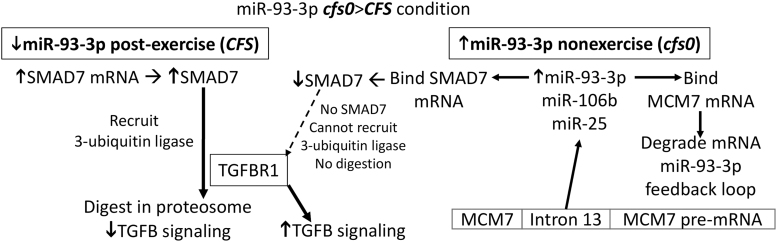
Proposed modulation of miR-93-3p, *MCM7*, SMAD7, and *TGFBR1* by exercise (***cfs0***>***CFS*** condition).

In the ***cfs0***>***CFS*** condition, miR-93-3p was elevated in nonexercise, and diminished after exercise. Elevated miR-93-3p in the nonexercise (***cfs0***) state binds SMAD7 mRNA leading to reduced SMAD7 protein (Fig. 5). As a result, 3-ubiquitin ligase cannot be recruited to degrade *TGFB1R1*, and so there is a relative activation of TGFB signaling. At the same time, the elevated miR-93-3p binds *MCM7* mRNA leading to its degradation. However, splicing of *MCM7* pre-mRNA releases intron 13 that is the source of miR-93-3p, mir-25, and miR-106b. miR-93-3p was diminished in the post-exercise (***CFS***) state, which may permit higher SMAD7 translation leading to recruitment of 3-ubiquitin ligase and degradation of *TGFBR1*. This conjecture requires verification *in vitro*.

#### Anatomical location

The quantile-normalized expression data from Tissue Atlas for miRNA^38^ showed that miR-200a-5p is abundant in cerebral cortex and white matter.

#### Brain

Brain regions containing the targets derived from DIANA (Table 3) were assayed using the ENRICHR tool^40^ and Jensen tissue database.^42^

The tissue that was most enriched with the 33 target genes was corpus callosum (*p*=0.00029). The relatively upregulated genes were *CCNE2*, *ELK4*, *HNRNPC*, *IQGAP1*, *MCM7*, *NCL*, *SCARB2*, *SCD*, *SETD7*, *SPOPL*, and *TCF3*. This list was interrogated in Pathway Studio, which identified the biological processes *Eat me signal: apoptotic cell induces phagocytosis*, *SIRT7 signaling in aging*, *Adherens junction assembly*, *Transcytosis*, *Cell cycle*, and *Metabolism of Glycerophospholipids and Ether Lipids.*

#### Choroid plexus

Because up to 70% of cerebrospinal fluid is produced in choroid plexus by ultrafiltration and secretion,^65^ we proposed that choroid plexus epithelial cells may be a major source of miRNAs in cerebrospinal fluid. The Allen brain atlas adult human brain tissue gene expression profile data set was reviewed, and a choroid plexus database was extracted and searched to find proteins that were upregulated or downregulated relative to whole brain (*p*<0.05). These agreed with a lateral ventricle database extracted from Jensen tissue atlas^42^ and text mining.

Targets that were overexpressed were *BAG6*, *EWSR1*, *IGF1R*, *IQGAP1*, *NUF1P2*, *PCBP2*, *PRPF8*, *PTPRJ*, *SIN3A*, and *TCF3*. DAVID identified *EWSR1*, *TCF3*, *IGF1*, and *IGF1R* in KEGG *transcriptional misregulation in cancer*. KEGG *Adherens junction* included *IQGAP1*, *IGF1R*, and *PTPRJ*. GO term RNA binding included *EWSR1*, *NUFIP2*, *PCBP2*, *PRPF8*, and *SIN3A*. Pathway Studio identified *IGF1R* as an important signaling mechanism in choroid plexus epithelium. These mechanisms may be accentuated in choroid plexus during the post-exercise ***CFS*** condition when miRNAs were diminished and targets would be anticipated to be abundantly expressed.

mRNAs that were downregulated (*p*≤0.05) in choroid plexus were *CCND2* (cell cycle), *CDC42* (adhesion), *FASN*, *DYNC1H1*, *FYN*, *HNRNPC*, *SCD*, and *SPOPL*. DAVID identified downregulated GO:0016020 membrane with *CDC42*, *FASN*, *DYNC1H1*, *HNRNPC*, and *SCD*. Focal adhesion (hsa 04510) was indicated by *CDC42*, *FYN*, and *CCND2*. Pathway Studio predicted downregulation of *leukocyte activation*, and *cell processes related to Adherens junctions*. Downregulated targets may be relevant to the *cfs0* condition when miRNAs were elevated and mRNAs and proteins would be anticipated to be repressed.

Targets that were equivalent between choroid plexus and whole brain were *TGFBR1*, *MCM7*, *ACTB*, *ASH1L*, *CCNE2*, *DCP2*, *DYRK1A*, *ELK4*, *H3F3B*, *KMT2D*, *NCL*, *SCARB2*, *SETD7*, *SUV420H1*, and *ZNF703.*

#### Brain cell types

Only 7 of 33 DIANA targets were matched to transcriptomes of individual brain cell types using Genecards.^44^ *IGF1R*, *TGFBR1*, *DYNC1H1*, and *IQGAP1* were matched to microglia. All seven IPA targets were localized to neurons, with six out of seven in astrocytes (RAP1B not identified). The seven targets were widely expressed in amygdala, corpus callosum, and cerebral cortex. Microglia contained ADCY3, MAP2K4, and RAP2A. The targets were anticipated to be downregulated in ***cfs0*** when miRNA levels were increased, and relatively upregulated after exercise (***cfs0***>***CFS*** miRNA condition). Oligodendrocytes expressed *TCF3*.^44^ Other targets were found in multiple lineages and were not enriched in any single cell type.

Subsets of our target genes were matched to the previously defined brain cell transcriptome modules CD1-neuron (*ACTB*, *BAG6*, *DYNC1H1*, *FASN*, and *PRPF8*) and CD5-neuron (*CCND2*, *CDC42*, and *DYNC1L1*).^66^

#### Metabolomics

Metabolomic alterations have been suggested in CFS.^67,68^ The list of target genes was searched in Human Metabolite Data Base (HMDB) using Metaboanalyst,^69^ which identified S-adenosylhomocysteine (HMDB00939), S-adenosylmethionine (HMDB01185), and manganese (HMDB01333) that participate in methane, glyoxylate, and amino acid metabolism pathways (*p*<0.025).

#### Treatment type

Drugs for the target proteins were identified through analysis in Ingenuity pathway software^®26^ (Supplementary Table S10). Oncology drugs targeted *FYN*, *IGF1R*, *TGFBR1*, and *FASN*.

## Discussion

The informatics search tools were based on literature text mining using proprietary algorithms. There was a surprising lack of overlap between the search in IPA, Pathway Studio, Cytoscape, and other tools. We placed priority on the DIANA and IPA searches that used combinations of miRNAs to identify gene intersection, proteins, and their pathways. The rationale was that single miRNAs can modulate hundreds of mRNAs. Therefore, we searched for targets that were regulated in unison by several of the miRNAs in the ***cfs0***>***CFS*** condition. This approach included weighting the pathways and targets to select those that were present in shared pathways such as PIK3R3, GNAQ, *IGFR1*, and *CDC42*. The union of the DIANA and IPA miRNA target lists consolidated the signaling mechanisms to (1) *GNAQ–PLC–MAPK*, (2) *IGF1R*, (3) *RAP1B*, (4) *PIK3R3-AKT*, (5) *JNK,* and (6) *CDC42–adhesion–extracellular matrix*. Pathways related to cell adhesion were a recurring theme from DIANA, KEGG, literature (Table 5), Cytoscape modules (Table 7), and STRING (Fig. 3C). Other targets were directed at *histone and chromatin modification*, *RNA processing* (e.g., Cytoscape module 2; Fig. 3B), and *cell cycle*. It was anticipated that analysis of the DIANA results would reproduce some pathways such as *Adherens junction*, but the iterative search process provided other pathways that may be of value to generate hypotheses of cellular dysfunction in ME/CFS and after exercise.

miR-200a-5p was elevated in the nonexercise specimens (***cfs0***>***CFS***) and provides links to *TGFBR1*, TGFB and adhesion. Elevated miR-200a/b levels decrease the expression of ZEB1 and SIP1, which in turn allow elevated E-cadherin expression that promotes an epithelial cell phenotype.^70^ Pathway Studio analysis predicted TGFB1 would inhibit miR-200a, which would promote upregulation of ZEB1 and SIP1, downregulation of the E-cadherin epithelial marker, and upregulation of the mesenchymal markers fibronectin and N-cadherin.^70^ This balancing interaction may be active in the choroid plexus.

The miRNA data were derived from cerebrospinal fluid expression patterns, which may have the greatest impact on gene regulation in choroid plexus, epithelial lining of the cerebral ventricles and associated immune cells, superficial subventricular gray and white matter, and floor of the fourth ventricle. Microglia and neurons were implicated in exercise-induced alterations of miRNAs and their patterns of target expression using GeneCards.

The high expression of the target genes in the corpus callosum was of interest because CFS patients have decreased white matter volumes by voxel-based morphometry.^71,72^ The affected white matter tracts extend from the inferior frontal lobe, dorsal right prefrontal lobe, bilateral internal and external capsules, and anterior midbrain, to the bilateral pons.

These outcomes are limited to the effects of the physiological stressor of exercise between two groups of ME/CFS subjects. The analysis was based on the exercise-induced changes in cerebrospinal fluid miRNAs in ME/CFS (***cfs0***>***CFS*** condition), but does not identify differences between ME/CFS and control status either before or after exercise. miRNA levels were equivalent between control, ME/CFS (***cfs0***), and Gulf War Illness (GWI) in the nonexercise (baseline) period.^9^ The pathways that were inferred for the ***cfs0***>***CFS*** condition are likely to be different from those found between nonexercise control and GWI subjects with their appropriate post-exercise comparison groups. Different cohorts were studied in cross-section. No subject had lumbar punctures both before (***cfs0***) and after (***CFS*)** exercise. This was a sample of convenience and not a case–control study.

A novel weighting strategy was used to find targets that were recognized in parallel by the combination of miRNAs (genes intersection). The combinations may have reduced false positive rates compared to conducting searches with individual miRNAs (gene union). The weighted approach may have excluded some targets that were selected by single miRNAs that had large effects in brain cells. Future studies are required to investigate *in vitro* and *in vivo* effects of both single miRNAs and the combination.

The literature searches for individual miRNAs did not reflect the complexity of the *in vivo* interactions between combinations of miRNAs, multiple gene targets, and pathways. The literature search did not verify the miRNA-mRNA interactions found using combinations of miRNAs with DIANA or IPA miRNA filter, suggesting that our weighting strategy had advantages over assessments with single overexpressed miRNAs in model systems.^49,51,52,55,56,60–62,64^

The categories of GO were often general and vague (e.g., organelle) and did not indicate more specific mechanisms. Therefore, secondary analysis of these clusters of targets was needed in other software such as DAVID.

IPA and Cytoscape used proprietary software tools and databases to find their lists of targets, but these also did not overlap with the DIANA or literature searches. The outcomes of these tools suggested not only direct inhibitory actions of the miRNAs on the 3′ UTR of mRNAs but also “positive” effects with increased expression of some mRNAs (Table 4) that may be the result of modulation of other regulatory mRNAs, transcription factors, target proteins, and their pathways (Fig. 4). These search strategies did not provide consistent probability estimates that could be used for additional connectivity and mechanistic investigations.^73^

Therapeutic implications were limited because many of the available drugs are indicated for cancer treatments, and have not been studied in neurological or other diseases. However, the proposed mechanisms (Fig. 4) may offer new insights for pathogenic mechanisms, experimental verification, and future drug treatments.

The miRNAs were measured in cerebrospinal fluid, and we do not have histological or single cell RNA evidence of their origins. Similarly, the means of secretion in exosomes or other microvesicles and target cells, and kinetics of intercellular regulation are not known. The postulated interactions of the combinations of miRNAs and effects on their targets and pathways will require confirmation *in vitro*. Future studies may use post-mortem human tissues or animal models to study miRNA production, transport, and the kinetics of effector functions in the baseline state and after exercise to better understand ME/CFS and post-exertional malaise.

## Supplementary Material

Supplemental data

## Supplementary Material

Supplementary Method

## Abbreviations Used

ANOVA: analysis of variance
Ct: cycle thresholds
FDR: false discovery rate
GO: Gene Ontology
GWI: Gulf War Illness
HSD: Honest Significant Difference
IPA: Ingenuity Pathway Analysis
KEGG: Kyoto Encyclopedia of Genes and Genomes
ME/CFS: myalgic encephalomyelitis/chronic fatigue syndrome
miRNA: micro-RNA
qPCR: quantitative polymerase chain reaction
ROC: receiver operating characteristics
