# Supplementary material for: Informatics Inference of Exercise-Induced Modulation of Brain Pathways Based on Cerebrospinal Fluid Micro-RNAs in Myalgic Encephalomyelitis/Chronic Fatigue Syndrome
Source: Netw Syst Med. 2020 Nov 18;3(1):142–58. doi: 10.1089/nsm.2019.0009 (PMC7703497; doi:10.1089/nsm.2019.0009)
Supplement: Supplemental data [file Supp_Data.docx]

**SUPPLEMENTARY ONLINE MATERIAL:**

**METHODS:**

**Table S1**: miRNA elevated in ***cfs0*** compared to ***CFS*** (***cfs0>CFS*** condition). ΔΔCt between pairs of groups indicating relative miRNA levels after normalization of QPCR using 11 miRNAs (N11) (mean ± SD). Differences were significant if ANOVA p<0.05, Tukey Honest Significant Difference (HSD) <0.05, FDR<0.05, and receiver operating characteristic asymptotic significance <0.05. Let-7i-5p was not significant by FDR for ***cfs0>CFS.***

| miRNA | ΔΔCt | HSD | FDR |
| --- | --- | --- | --- |
| miR-608 | 2.85±2.43 | HSD=0.001 | FDR=0.0037 |
| miR-328 | 4.61±3.67 | HSD=0.000 | FDR=0.0013 |
| miR-200a-5p | 2.53±2.16 | HSD=0.001 | FDR=0.0036 |
| miR-93-3p | 2.10±2.03 | HSD=0.001 | FDR=0.015 |
| miR-92a-3p | 2.29±2.06 | HSD=0.008 | FDR=0.0070 |
| let-7i-5p | 1.91±2.25 | HSD=0.034 | FDR=0.080 |

**Analysis of miRNA targets from DIANA mirPath** ^17^

The combination of miRNAs was entered into DIANA miRpath v3.0 ^17^ software and DIANA-TarBase v7.0 database ^72^ to identify the intersection of pathways from KEGG (Kyoto Encyclopedia of Genes and Genomes) ^25^ and GO (Gene Ontology) ^26^ databases using the highest number of miRNAs for each search . Targets were found by weighting KEGG and GO outputs because target genes appear in multiple pathways and may be affected by multiple miRNAs.

Selection criteria include:

1. Maximum number of miRNAs in search
2. Intersection of pathways between miRNAs to select possible addition or segregation effects.
3. Pathways with significance (p<0.001) chosen here. Select your preferred p-value.

These criteria were introduced to select the highest probability targets present in most pathways since in reality the number of pathways will be many and thousands of genes will be presented as targets for the miRNA list.

**Steps to find the target genes from DIANA mirPath v3.0 for the given list of miRNAs:**

- Input the list of miRNAs in the mirPathv.3 tool. Choose KEGG pathways analysis.
- Choose gene intersection.
- Choose a threshold p value for finding the significant pathways (p <0.001) and Fisher’s Exact test for enrichment analysis method.
- Copy the miRpath v3.0 results to an excel table. (Table S2)

Example: **Table S2:** Example of DIANA output

| # | KEGG pathway | p-value | #genes | #miRNAs |
| --- | --- | --- | --- | --- |
| 1 | Fatty acid biosynthesis (hsa00061) | 3.12E-36 | 1 | 2 |
| 2 | Fatty acid metabolism (hsa01212) | 2.46E-13 | 2 | 2 |
| 3 | Adherens junction (hsa04520) | 0.000255854 | 7 | 4 |
| 4 | Lysine degradation (hsa00310) | 0.000485208 | 4 | 3 |
| 5 | Transcriptional misregulation in cancer (hsa05202) | 0.000514768 | 7 | 4 |

- Download the genes for each pathway.
- Find the number of KEGG pathways that expressed each gene (#KEGG).
- Calculate weight for each gene using the formula weight= (number of miRNA) *(-logP)

**Table S3:** Genes from DIANA target list arranged in alphabetical order. Genes and the KEGG pathways it appeared (Table S2) are given in this table. *FASN* and *IGF1R* appeared in two KEGG pathways and hence their weight must be summed up .

| #KEGG pathway number | Gene | Weight |
| --- | --- | --- |
| 3 | *ACTB* | 14.36803 |
| 4 | *ASH1L* | 9.942215 |
| 5 | *CCND2* | 13.15355 |
| 3 | *CDC42* | 14.36803 |
| 5 | *ELK4* | 13.15355 |
| 5 | *EWSR1* | 13.15355 |
| 1 | *FASN* | 71.01051 |
| 2 | *FASN* | 25.2178 |
| 3 | *FYN* | 14.36803 |
| 5 | *H3F3B* | 13.15355 |
| 3 | *IGF1R* | 14.36803 |
| 5 | *IGF1R* | 13.15355 |
| 3 | *IQGAP1* | 14.36803 |
| 4 | *KMT2D* | 9.942215 |
| 3 | *PTPRJ* | 14.36803 |
| 2 | *SCD* | 25.2178 |
| 4 | *SETD7* | 9.942215 |
| 5 | *SIN3A* | 13.15355 |
| 4 | *SUV420H1* | 9.942215 |
| 5 | *TCF3* | 13.15355 |
| 3 | *TGFBR1* | 14.36803 |

- Find the number of KEGG pathways that expressed each gene (#KEGG).
- Use the MATLAB script given below to calculate the sum of weights and # of times a gene appeared. because in reality number of significant pathways will be higher and thousands of genes will be presented as targets for the list of miRNA from DIANA miRpath.

**To use the MATLAB code:**

- Create an excel sheet with the following columns created above from DIANA miRpath.

Example: **Table S4:** Example for creating a table to input into the MATLAB code.

| #KEGG pathway number | Gene | Weight |
| --- | --- | --- |
| 3 | *ACTB* | 14.36803 |
| 4 | *ASH1L* | 9.942215 |
| 5 | *CCND2* | 13.15355 |
| 3 | *CDC42* | 14.36803 |
| 5 | *ELK4* | 13.15355 |
| 5 | *EWSR1* | 13.15355 |
| 1 | *FASN* | 71.01051 |
| 2 | *FASN* | 25.2178 |
| 3 | *FYN* | 14.36803 |
| 5 | *H3F3B* | 13.15355 |
| 3 | *IGF1R* | 14.36803 |
| 5 | *IGF1R* | 13.15355 |
| 3 | *IQGAP1* | 14.36803 |
| 4 | *KMT2D* | 9.942215 |
| 3 | *PTPRJ* | 14.36803 |
| 2 | *SCD* | 25.2178 |
| 4 | *SETD7* | 9.942215 |
| 5 | *SIN3A* | 13.15355 |
| 4 | *SUV420H1* | 9.942215 |
| 5 | *TCF3* | 13.15355 |
| 3 | *TGFBR1* | 14.36803 |

Name the excel file (example:vk_cfs.xlsx)

**In MATLAB:**

- Import the excel sheet (**Table S5**) and the script (given below).
- Enter the command in the command window: computeWeight (‘excel file name’, column number of weight, column number of gene name);
- Example: computeWeight (‘vk_cfs.xlsx’, 2, 3)
- MATLAB output will be created with the sum of weights and number of times a gene appeared for each gene.

Example: **Table S5:** Example for excel sheet to import into MATLAB. Genes with their summed-up weights and summed up number of times they appeared are given in this table.

| Gene | Sum of Weight | Sum of hits |
| --- | --- | --- |
| *ACTB* | 14.36803 | 1 |
| *ASH1L* | 9.942215 | 1 |
| *CCND2* | 13.15355 | 1 |
| *CDC42* | 14.36803 | 1 |
| *ELK4* | 13.15355 | 1 |
| *EWSR1* | 13.15355 | 1 |
| *FASN* | 96.22831 | 2 |
| *FYN* | 14.36803 | 1 |
| *H3F3B* | 13.15355 | 1 |
| *IGF1R* | 27.52159 | 2 |
| *IQGAP1* | 14.36803 | 1 |
| *KMT2D* | 9.942215 | 1 |
| *PTPRJ* | 14.36803 | 1 |
| *SCD* | 25.2178 | 1 |
| *SETD7* | 9.942215 | 1 |
| *SIN3A* | 13.15355 | 1 |
| *SUV420H1* | 9.942215 | 1 |
| *TCF3* | 13.15355 | 1 |
| *TGFBR1* | 14.36803 | 1 |

- To choose the target genes list sort the output table by weight and also by the # of pathways per gene.
- Select a threshold at a breakpoint in the two lists that optimizes the number of targets and their weights. Scree plot can also be drawn to select breakpoints if there are large numbers of candidate genes. The breakpoint will be usually about 20% to 30% of the list of potential targets. Analysis of scree plots suggest the breakpoint can be chosen for the above output table between 71.01 and 7.204 weight.
- Repeat the same for GO category in DIANA miRpath and find the weighted genes list.
- Then reconcile the KEGG and GO list by removing the duplicates between them.
- Final list of gene targets for the given list of miRNA is obtained.

**MATLAB script for finding out the weighted genes from DIANA mirPath v3.0:**

function computeWeight(fName, wCol, geneCol)

% Read excel file

(~, ~, data) = xlsread(fName);

% Find number rows of data to be processed

tmp = data(2:end, wCol);

for i=1:numel(tmp)

if(isnan(tmp{i}))

else

s=i;

end

end

% Read pathway number, weight and gene name

weight = data(2:2+s-1, wCol);

geneList = data(2:2+s-1, geneCol);

% Computer sum of weights

eWeight=zeros(numel(geneList),1);

cntHits=zeros(numel(geneList),1);

for gcount=1:numel(geneList)

gene = geneList{gcount};

for i=1:numel(geneList)

if(strcmp(char(geneList{i}),gene))

eWeight(gcount)=eWeight(gcount)+double(weight{i});

cntHits(gcount)=cntHits(gcount)+1;

end

end

end

gL = geneList;

eW = eWeight;

cH = cntHits;

% Empty duplicate rows

for gcount=1:numel(geneList)

gene = geneList{gcount};

if(~isempty(geneList{gcount}))

for i=1:numel(geneList)

if(strcmp(char(geneList{i}),gene))

if(gcount~=i)

geneList{i}=();

end

end

end

end

end

% Remove duplicate rows

resData{numel(geneList),3}=();

valData=0;

for gcount=1:numel(geneList)

if(~isempty(geneList{gcount}))

valData=valData+1;

resData{valData,1}=geneList{gcount};

resData{valData,2}=eWeight(gcount);

resData{valData,3}=cntHits(gcount);

end

end

resData(valData:end, :)=();

% Remove duplicate rows

rD{numel(gL),3}=();

valData=0;

for gcount=1:numel(gL)

valData=valData+1;

rD{valData,1}=gL{gcount};

rD{valData,2}=eW(gcount);

rD{valData,3}=cH(gcount);

end

% Save results

xlswrite(('out_', fName), resData);

end

**Table S6:** DIANA-mirPath outcomes for KEGG pathways (12). P-value < 0.01 was considered for significance. #of genes in the pathways with using #miRNAs =2. Weight was calculated using the formula: #miRNAs (-log p value). Pathways with p-value>0.01 were excluded from analysis.

| # | KEGG pathway | p-value | #genes | #miRNAs | Weight |
| --- | --- | --- | --- | --- | --- |
| 1 | Fatty acid biosynthesis (hsa00061) | 3.12E-36 | 1 | 2 | 71.01051 |
| 2 | Fatty acid metabolism (hsa01212) | 2.46E-13 | 2 | 2 | 25.2178 |
| 3 | Adherens junction (hsa04520) | 0.000256 | 7 | 4 | 14.36803 |
| 4 | Lysine degradation (hsa00310) | 0.000485 | 4 | 3 | 9.942215 |
| 5 | Transcriptional misregulation in cancer (hsa05202) | 0.000515 | 7 | 4 | 13.15355 |
|  |  |  |  |  |  |
| 6 | Viral myocarditis (hsa05416) | 0.003162 | 7 | 4 | 10.00035 |
| 7 | Cell cycle (hsa04110) | 0.005299 | 11 | 4 | 9.103179 |
| 8 | Oocyte meiosis (hsa04114) | 0.006567 | 7 | 3 | 6.54782 |
| 9 | RNA transport (hsa03013) | 0.00813 | 10 | 4 | 8.359669 |
| 10 | Salmonella infection (hsa05132) | 0.023509 | 7 | 4 | 6.515075 |
| 11 | Thyroid hormone signaling pathway (hsa04919) | 0.024143 | 9 | 4 | 6.46882 |
| 12 | Shigellosis (hsa05131) | 0.028625 | 6 | 3 | 4.629747 |
| 13 | Bacterial invasion of epithelial cells (hsa05100) | 0.029554 | 4 | 3 | 4.588143 |
| 14 | Chronic myeloid leukemia (hsa05220) | 0.044437 | 5 | 4 | 5.40904 |
| 15 | Pathways in cancer (hsa05200) | 0.049946 | 15 | 4 | 5.205984 |

**Table S7**: DIANA-mirPath outcomes for GO categories (12). P-value < 0.01 was considered for significance. #of genes in the pathways with using #miRNAs =4. Weight was calculated using the formula: #miRNAs (-log p value). 60 total GO categories with 57 having p>0.001 that were not considered significant for this strategy.

| # GO | GO Category | p-value | #genes | #miRNAs | Weight |
| --- | --- | --- | --- | --- | --- |
| 1 | nucleoplasm (GO:0005654) | 8.19E-05 | 8 | 4 | 16.34773 |
| 2 | ribonucleoprotein complex (GO:0030529) | 0.00067 | 5 | 4 | 12.69678 |
| 3 | organelle (GO:0043226) | 0.00067 | 16 | 4 | 12.69678 |
|  |  |  |  |  |  |
| 4 | mitotic cell cycle (GO:0000278) | 0.001986 | 4 | 3 | 8.105754 |
| 5 | cytosol (GO:0005829) | 0.001986 | 9 | 4 | 10.80767 |
| 6 | cellular component assembly (GO:0022607) | 0.004705 | 6 | 4 | 9.309818 |
| 7 | gene expression (GO:0010467) | 0.005474 | 4 | 4 | 9.046915 |
| 8 | RNA binding (GO:0003723) | 0.005592 | 7 | 4 | 9.0096 |
| 9 | cellular nitrogen compound metabolic process (GO:0034641) | 0.005626 | 10 | 4 | 8.999157 |
| 10 | exoribonuclease activity, producing 5'-phosphomonoesters (GO:0016896) | 0.009322 | 1 | 3 | 6.09147 |
| 11 | cytoplasmic ribonucleoprotein granule (GO:0036464) | 0.009322 | 2 | 4 | 8.121959 |
| 12 | DNA replication initiation (GO:0006270) | 0.009457 | 2 | 3 | 6.072785 |
| 13 | ATP-dependent chromatin remodeling (GO:0043044) | 0.009457 | 2 | 4 | 8.097047 |
| 14 | mRNA splicing, via spliceosome (GO:0000398) | 0.01179 | 3 | 4 | 7.71398 |
| 15 | nucleosomal DNA binding (GO:0031492) | 0.011908 | 2 | 4 | 7.696675 |
| 16 | enzyme binding (GO:0019899) | 0.017401 | 5 | 4 | 7.037739 |
| 17 | small conjugating protein binding (GO:0032182) | 0.018047 | 2 | 3 | 5.230786 |
| 18 | poly(A) RNA binding (GO:0044822) | 0.018047 | 6 | 4 | 6.974382 |
| 19 | RNA splicing (GO:0008380) | 0.019151 | 3 | 4 | 6.871272 |
| 20 | dynein light intermediate chain binding (GO:0051959) | 0.023481 | 1 | 3 | 4.887856 |
| 21 | U5 snRNA binding (GO:0030623) | 0.029719 | 1 | 3 | 4.580903 |
| 22 | positive regulation of transcription of nuclear large rRNA transcript from RNA polymerase I promoter (GO:1901838) | 0.029719 | 1 | 3 | 4.580903 |
| 23 | RNA polymerase II distal enhancer sequence-specific DNA binding (GO:0000980) | 0.029719 | 2 | 4 | 6.107871 |
| 24 | negative regulation of polyamine transmembrane transport (GO:1902268) | 0.030067 | 1 | 3 | 4.565729 |
| 25 | protein complex (GO:0043234) | 0.030067 | 8 | 4 | 6.087638 |
| 26 | cell junction organization (GO:0034330) | 0.032228 | 2 | 4 | 5.967071 |
| 27 | ribonucleoprotein complex assembly (GO:0022618) | 0.033321 | 2 | 4 | 5.909135 |
| 28 | negative regulation of homotypic cell-cell adhesion (GO:0034111) | 0.036074 | 1 | 3 | 4.3284 |
| 29 | oocyte growth (GO:0001555) | 0.036074 | 1 | 3 | 4.3284 |
| 30 | m7G(5')pppN diphosphatase activity (GO:0050072) | 0.036074 | 1 | 3 | 4.3284 |
| 31 | positive regulation of protein deacetylation (GO:0090312) | 0.036074 | 1 | 3 | 4.3284 |
| 32 | cell cycle (GO:0007049) | 0.036074 | 4 | 3 | 4.3284 |
| 33 | mRNA metabolic process (GO:0016071) | 0.036092 | 2 | 3 | 4.327766 |
| 34 | ornithine decarboxylase inhibitor activity (GO:0008073) | 0.039073 | 1 | 3 | 4.224382 |
| 35 | proteasome-mediated ubiquitin-dependent protein catabolic process (GO:0043161) | 0.039073 | 2 | 3 | 4.224382 |
| 36 | nucleus (GO:0005634) | 0.039073 | 14 | 4 | 5.632509 |
| 37 | G1/S transition of mitotic cell cycle (GO:0000082) | 0.040134 | 2 | 3 | 4.189472 |
| 38 | positive regulation of mammary gland epithelial cell proliferation (GO:0033601) | 0.040863 | 1 | 3 | 4.16601 |
| 39 | BAT3 complex (GO:0071818) | 0.040863 | 1 | 3 | 4.16601 |
| 40 | mRNA processing (GO:0006397) | 0.041723 | 3 | 4 | 5.518483 |
| 41 | catabolic process (GO:0009056) | 0.041847 | 5 | 4 | 5.513337 |
| 42 | internal peptidyl-lysine acetylation (GO:0018393) | 0.045414 | 1 | 3 | 4.02842 |
| 43 | Tat protein binding (GO:0030957) | 0.045414 | 1 | 3 | 4.02842 |
| 44 | tail-anchored membrane protein insertion into ER membrane (GO:0071816) | 0.045414 | 1 | 3 | 4.02842 |
| 45 | spliceosomal complex (GO:0005681) | 0.045414 | 2 | 4 | 5.371227 |
| 46 | chromatin modification (GO:0016568) | 0.045414 | 2 | 4 | 5.371227 |
| 47 | U5 snRNP (GO:0005682) | 0.045865 | 1 | 3 | 4.015567 |
| 48 | adherens junction assembly (GO:0034333) | 0.045865 | 1 | 3 | 4.015567 |
| 49 | catalytic step 2 spliceosome (GO:0071013) | 0.045865 | 2 | 4 | 5.354089 |
| 50 | polysomal ribosome (GO:0042788) | 0.046813 | 1 | 3 | 3.98891 |
| 51 | U6 snRNA binding (GO:0017070) | 0.047681 | 1 | 3 | 3.96497 |
| 52 | negative regulation of defense response to virus (GO:0050687) | 0.047681 | 1 | 3 | 3.96497 |
| 53 | focal adhesion (GO:0005925) | 0.047681 | 3 | 4 | 5.286627 |
| 54 | chlorophyll biosynthetic process (GO:0015995) | 0.048388 | 1 | 3 | 3.945775 |
| 55 | magnesium chelatase activity (GO:0016851) | 0.048388 | 1 | 3 | 3.945775 |
| 56 | MLL5-L complex (GO:0070688) | 0.048388 | 1 | 3 | 3.945775 |
| 57 | polyamine biosynthetic process (GO:0006596) | 0.048388 | 1 | 3 | 3.945775 |
| 58 | photosynthesis (GO:0015979) | 0.048388 | 1 | 3 | 3.945775 |
| 59 | tau protein binding (GO:0048156) | 0.049146 | 1 | 3 | 3.925533 |
| 60 | positive regulation of intracellular estrogen receptor signaling pathway (GO:0033148) | 0.049878 | 1 | 3 | 3.906273 |

**Table S8**: KEGG pathways identified by DAVID ^29^ for the list of targets from IPA MicroRNA Target Filter ^®28^.

| KEGG_PATHWAY | Count | Genes | P-Value | Benjamini |
| --- | --- | --- | --- | --- |
| GnRH signaling pathway | 4 | GNAQ,ADCY3,CAMK2A,MAP2K4 | 2.20E-05 | 2.40E-03 |
| Inflammatory mediator regulation of TRP channels | 4 | GNAQ,ADCY3,CAMK2A,PIK3R3 | 2.70E-05 | 1.50E-03 |
| Cholinergic synapse | 4 | GNAQ,ADCY3,CAMK2A,PIK3R3 | 4.00E-05 | 1.50E-03 |
| Platelet activation | 4 | GNAQ,RAP1B,ADCY3,PIK3R3 | 6.40E-05 | 1.70E-03 |
| Rap1 signaling pathway | 4 | GNAQ,RAP1B,ADCY3,PIK3R3 | 2.70E-04 | 4.90E-03 |

**Table S9:** Transcription factors that regulate parent genes and their miRNAs ^74^. MYC which is a regulator for miR-93 and miR-92A is found in all three targets. ELK4 is one of the targets found by DIANA mirPath target analysis (Table 3).

| Parent genes | ELMO3 | MCM7 | SEMA4G |
| --- | --- | --- | --- |
| Transcription factors | \| ARNT \| \| --- \| \| ATF2 \| \| ATOH1 \| \| BARHL1 \| \| BCL6 \| \| CREB1 \| \| CTCF \| \| CTCFL \| \| E2F6 \| \| EHF \| \| ELF1 \| \| ESR1 \| \| ETV1 \| \| FOXD2 \| \| GMEB2 \| \| HIF1A \| \| HOXC6 \| \| KDM5B \| \| KLF4 \| \| KLF5 \| \| MAX \| \| MAZ \| \| MBD2 \| \| MYC \| \| NANOG \| \| NR2F1 \| \| NR2F2 \| \| RFX5 \| \| SNAI2 \| \| SPDEF \| \| SRF \| \| TCF12 \| \| TCF4 \| \| TFAP2C \| \| TFAP4 \| \| USF1YY1 \| \| ZBTB7A \| \| ZNF143 \| \| ZNF552 \| \| ZNF84 \| | \| ARNT \| \| --- \| \| ATF2 \| \| ATF3 \| \| BARX2 \| \| BCLAF1 \| \| BHLHE40 \| \| CEBPB \| \| CREB1 \| \| E2F1 \| \| E2F4 \| \| E2F6 \| \| ELF1 \| \| ELK4 \| \| ERG \| \| ETS1 \| \| FOS \| \| GABPA \| \| GLI2 \| \| HNF4G \| \| HOXC6 \| \| IRF3 \| \| KDM5A \| \| KLF4 \| \| KLF5 \| \| LEF1 \| \| MAX \| \| MTA3 \| \| MYBL2 \| \| MYC \| \| MYOD1 \| \| MZF1 \| \| NANOG \| \| NFE2 \| \| NFIC \| \| NFYA \| \| PAX5 \| \| PBX3 \| \| RELA \| \| RFX2 \| \| RUNX3 \| \| SNAI2 \| \| SP1 \| \| SP2 \| \| SPDEF \| \| STAT3 \| \| TBP \| \| TFAP2C \| \| USF1 \| \| USF2 \| \| YY1 \| \| ZBTB7A \| \| ZFP42 \| \| ZNF384 \| \| ZNF84 \| | \| AR \| \| --- \| \| ARID3A \| \| ARNT \| \| BCL6 \| \| BHLHE40 \| \| CDX2 \| \| CTCF \| \| CTCFL \| \| E2F6 \| \| EGR1 \| \| GABPA \| \| HIF1A \| \| HINFP \| \| HNF4A \| \| HNF4G \| \| IRF1 \| \| KDM5B \| \| KLF4 \| \| KLF9 \| \| MAX \| \| MAZ \| \| MBD4 \| \| MXI1 \| \| MYC \| \| MYOD1 \| \| NANOG \| \| NFIC \| \| NR5A1 \| \| POU5F1 \| \| RCOR1 \| \| SNAI2 \| \| SRF \| \| STAT1 \| \| TBP \| \| TP53 \| \| UBTF \| \| YY1 \| \| ZBTB7A \| \| ZNF143 \| \| ZNF552 \| |

**Table S10:** Drugs affecting the target proteins found from Ingenuity Pathway Analysis software® ^28^.

| Gene | Gene name | Location | Type | Drugs |
| --- | --- | --- | --- | --- |
| *FYN* | FYN proto-oncogene, Src family tyrosine kinase | Plasma Membrane | kinase | Dasatinib, JNJ-26483327 |
| *TGFBR1* | transforming growth factor beta receptor 1 | Plasma Membrane | kinase | Galunisertib, TEW-7197 |
| *IGF1R* | insulin like growth factor 1 receptor | Plasma Membrane | transmembrane receptor | Picropodophyllin, Linsitinib, Cixutumumab, Ganitumab, AVE1642, BMS-754807, XL228, BIIB022, IGF1, Dalotuzumab, Ceritinib, Brigatinib, (I-124)-CPD-1028, MM-141, IGF-methotrexate conjugate, PL225B, KW-2450, IGF-1R inhibitor |
| *FASN* | fatty acid synthase | Cytoplasm | enzyme | TVB-2640, Orlistat, Cerulenin, 3-V bioscience-2640 |

**Table S11:** miRNA-gene interactions from tarbase v8.0 ^72^ from DIANA tools.

| ARRDC3 | FNIP2 | MAP2K4 |
| --- | --- | --- |
| CD69 | GRAMD1B | MIA3 |
| DKK3 | ITGA5 | PPP1R37 |
| ERGIC2 | KLF4 | PTAR1 |
| FBXW7 | KLHDC10 | TEF |
| FMN2 | MAN2A1 | UBE2W |

**Table S12:** miRNA-gene interactions from microTCDS ^73^ from DIANA tool.

| **miR-328** | **miR-608** | **miR-200a-5p** | **miR-92a-3p** | **miR-93-3p** |
| --- | --- | --- | --- | --- |
| CA12 | SETD5 | WHSC1 | CD69 | FOXP2 |
| CFH | GTDC1 | RAB1A | FNIP1 | MED15 |
| ITGA5 | MAP3K13 | ZNF141 | PPP1R37 | DST |
| SNRK | FCRLA | ZNF396 | FAAH2 | PAXIP1 |
| FUZ | PALLD | ZNF738 | HIPK3 | GOLGB1 |
| FUBP1 | TMEM61 | CCDC144NL | SLC12A5 | NFAT5 |
| RPS6KA1 | THRA | NPIPA7 | PTAR1 | C11orf82 |
| FAM215A | NOVA2 | SUZ12 | FBXW7 | RP11-123K3.4 |
| ZNF430 | RBM25 | ZNF91 | BAZ2B | SUPT20H |
| ARID4B | DNAH12 | ZNF254 | ST6GAL2 | PHC1 |
| KIF20A | RP11-368I7.4 | ZNF708 | MYCBP2 | SLC35G3 |
| ETV6 | CDSN | RP11-758M4.1 | MAP2K4 | AC008394.1 |
| TCN2 | NUMBL | EPHA5 | UBE2W | GLCCI1 |
| SLC39A5 | NIT2 | ZNF493 | RGS3 | NOLC1 |
| RSBN1L | STX1B | ZNF714 | KLF4 | REPS1 |
| TRMT2A | LRRC3B | GLTSCR1 | ADRB1 | STXBP6 |
| EN2 | INA | ARMC9 | SGK3 | NEGR1 |
|  | ZNF415 | POLA1 | ITGA5 | KMT2C |
|  | ZMYND11 | SP3 | SOX4 | POU2F1 |
|  | NUTM2D | HMGB1 | TEF | DAPK1 |
|  | GATA5 | NR4A1 | CIC | PHTF1 |
|  | PRB2 | STK38L | PCDH11X | SKIV2L2 |
|  | DMWD | LARGE | PDZD2 | INO80 |
|  | EIF2AK2 | ZNF92 | PCDH11Y | PHLDB1 |
|  | GNG4 | ZBED5 | SRPR | TUBB6 |
|  | SYP | SRSF1 | ARRDC3 | DOCK5 |
|  | MICB | UBR3 | DSCAML1 | TARDBP |
|  | ZNF208 |  | MYO1B | MYLK4 |
|  | PNMA3 |  | ADAMTSL1 | STRC |
|  |  |  | WASL | NOVA1 |
|  |  |  |  | ATP6V1G2-DDX39B |
|  |  |  |  | AEBP2 |
|  |  |  |  | EAF2 |
|  |  |  |  | SGCE |
|  |  |  |  | PLCL1 |
|  |  |  |  | SIK3 |
|  |  |  |  | MTUS2 |
|  |  |  |  | MYO18A |
|  |  |  |  | PPP3CA |
|  |  |  |  | PCDH10 |
|  |  |  |  | NPIPB5 |
|  |  |  |  | PPP1R13B |
|  |  |  |  | FAM71F1 |
|  |  |  |  | SYNJ1 |
|  |  |  |  | DPP4 |
|  |  |  |  | MUC19 |
|  |  |  |  | GLI2 |
|  |  |  |  | NFIA |
|  |  |  |  | CCDC6 |

**FIGURES:**

**Figure S1**. Receiver operating characteristics of normalized ΔCt values were significantly different for the ***cfs0>CFS*** condition for miR-328, miR-608, miR-200a-5p, miR-93-3p and miR-92a-3p.

| miR-328 | miR-608 | miR-200a-5p |
| --- | --- | --- |
| 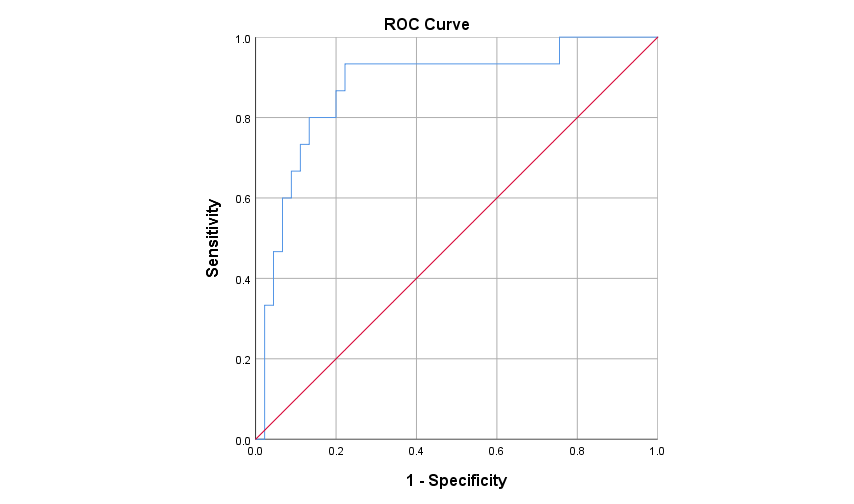 | 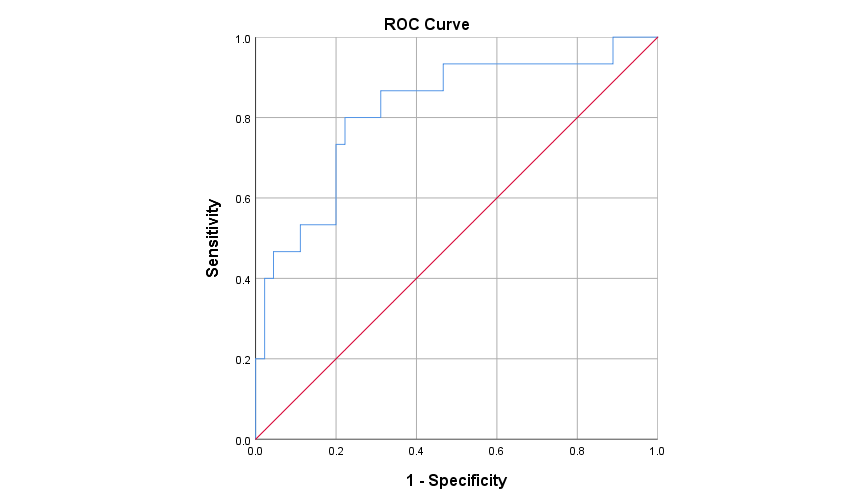 | 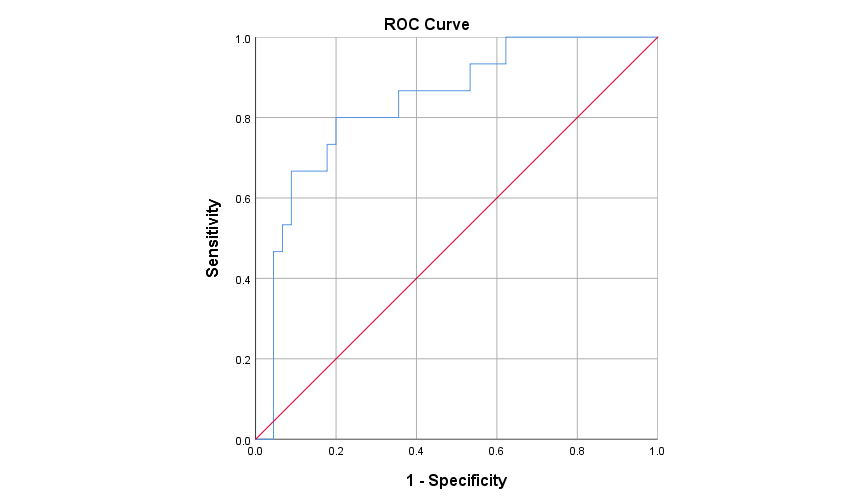 |
| miR-93-3p | miR-92a-3p |  |
| 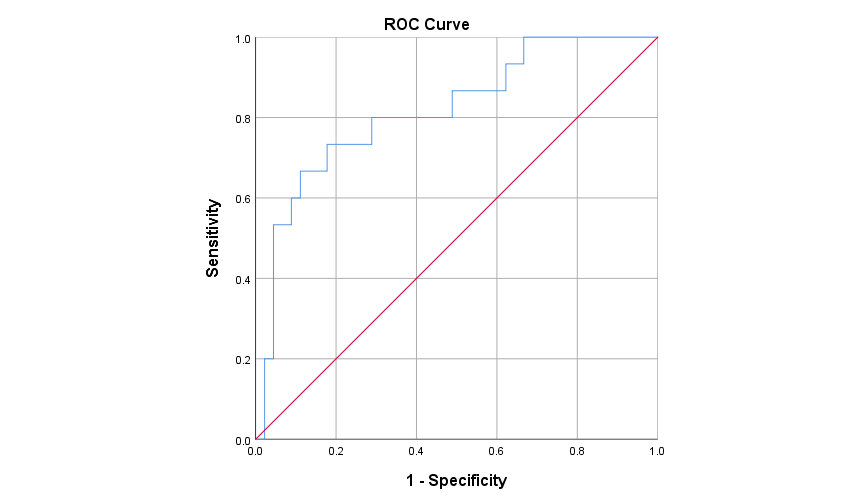 | 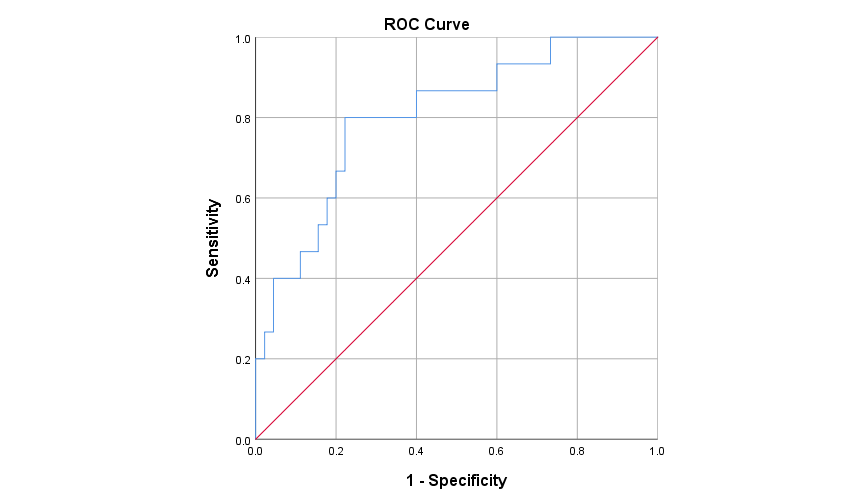 |  |

**REFERENCES:**

17. Vlachos IS, Zagganas K, Paraskevopoulou MD, et al. DIANA-miRPath v3.0: Deciphering microRNA function with experimental support. Nucleic Acids Res 2015;43:W460–W466.

25. Kanehisa M, Sato Y, Furumichi M, et al. New approach for understanding genome variations in KEGG. Nucleic Acids Res 2019;47:D590–D595.

26. Ontology CTG, Ashburner M, Ball CA, et al. The Gene Ontology Consortium, Michael Ashburner1, Catherine A. Ball3, Judith A. Blake4, David Botstein3, Heather Butler1, J. Michael Cherry3, Allan P. Davis4, Kara Dolinski3, Selina S. Dwight3, Janan T. Eppig4, Midori A. Harris3, David P. Hill4, Laurie Is. Nat Genet 2000;25:25–29.

28. Krämer A, Green J, Pollard J, et al. Causal analysis approaches in ingenuity pathway analysis. Bioinformatics 2014;30:523–530

29. Huang DW, Sherman BT, Lempicki RA. Systematic and integrative analysis of large gene lists using DAVID bioinformatics resources. Nat Protoc 2009;4:44–57.

72.Karagkouni D, Paraskevopoulou D, Hatzigeorgiou AG, et al. DIANA-TarBase v8: a decade-long collection of experimentally supported miRNA–gene interactions, Nucleic Acids Res. 2013;46(Database issue): D239–D245.

73.Paraskevopoulou MD, Georgakilas G, Vlachos IS, et al. DIANA-microT web server v5.0: service integration into miRNA functional analysis workflows. Nucleic Acids Res. 2013;41(Web Server issue):W169-7

74. Zhou KR, Liu S, Sun WJ, Zheng LL, Zhou H, Qu LH et al., (2017). ChIPBase v2.0: decoding transcriptional regulatory networks of non-coding RNAs and protein-coding genes from ChIP-seq data. Nucleic Acids Res.45(D1): D43-D50.
